# Charge Dynamics Electron Microscopy: Nanoscale Imaging of Femtosecond Plasma Dynamics

**DOI:** 10.1021/acsnano.2c10482

**Published:** 2023-02-13

**Authors:** Ivan Madan, Eduardo J. C. Dias, Simone Gargiulo, Francesco Barantani, Michael Yannai, Gabriele Berruto, Thomas LaGrange, Luca Piazza, Tom T. A. Lummen, Raphael Dahan, Ido Kaminer, Giovanni Maria Vanacore, F. Javier García de Abajo, Fabrizio Carbone

**Affiliations:** † Institute of Physics, École Polytechnique Fédérale de Lausanne, Lausanne1015, Switzerland; ‡ ICFO-Institut de Ciencies Fotoniques, The Barcelona Institute of Science and Technology, Castelldefels, Barcelona08860, Spain; ¶ Department of Quantum Matter Physics, University of Geneva, Geneva1211, Switzerland; § Department of Electrical and Computer Engineering, Technion Israel Institute of Technology, Haifa32000, Israel; ∥ BSSE Single Cell Facility, ETH Zurich, Basel4058, Switzerland; ⊥ Department of Materials Science, University of Milano-Bicocca, Milano20126, Italy; # ICREA, Institució Catalana de Recerca i Estudis Avançats, Barcelona08010, Spain

**Keywords:** Transmission electron microscopy, plasma dynamics, THz fields, nanoscale imaging, ultrafast dynamics

## Abstract

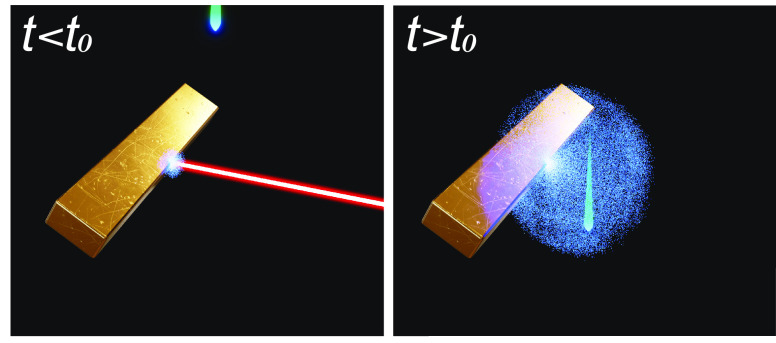

Understanding and actively controlling the spatiotemporal dynamics of nonequilibrium electron clouds is fundamental for the design of light and electron sources, high-power electronic devices, and plasma-based applications. However, electron clouds evolve in a complex collective fashion on the nanometer and femtosecond scales, producing electromagnetic screening that renders them inaccessible to existing optical probes. Here, we solve the long-standing challenge of characterizing the evolution of electron clouds generated upon irradiation of metallic structures using an ultrafast transmission electron microscope to record the charged plasma dynamics. Our approach to charge dynamics electron microscopy (CDEM) is based on the simultaneous detection of electron-beam acceleration and broadening with nanometer/femtosecond resolution. By combining experimental results with comprehensive microscopic theory, we provide a deep understanding of this highly out-of-equilibrium regime, including previously inaccessible intricate microscopic mechanisms of electron emission, screening by the metal, and collective cloud dynamics. Beyond the present specific demonstration, the here-introduced CDEM technique grants us access to a wide range of nonequilibrium electrodynamic phenomena involving the ultrafast evolution of bound and free charges on the nanoscale.

Modern ultrafast spectroscopy and microscopy strive to explore how electronic and crystal structures evolve on time scales of a few femtoseconds.^1−3^ However, the complex spatiotemporal dynamics of charge carriers photoexcited/emitted from surfaces has so far remained largely inaccessible because of the intrinsic difficulty of simultaneously addressing the nanometer and femtosecond scales on which the associated charge and transient near-field dynamics takes place. Understanding such dynamics is essential for the exploration of previously inaccessible physics and the development of applications in high-brightness electron sources in wake-field accelerators^4^ and RF/THz-driven emitters,^5−7^ ultrafast power electronics,^8^ plasma X-rays sources,^9−13^ plasma tailoring for photon down-conversion,^14^ and nuclear reactions in laser-generated plasma environments.^15,16^ In these contexts, the evolution of plasma is commonly monitored through far-field radiation, and some of its properties are inferred by comparison to numerical simulations,^5,17^ with no direct access into microscopic charge or field dynamics on their natural ultrafast nanoscopic scale. An exemplary scenario involving complex charge dynamics is the ensuing electron cloud emission from a solid target upon irradiation by high-fluence femtosecond laser pulses (see Figure 1a). The emitted electrons evolve by following distinct stages after light absorption: electron emission, expansion, deceleration, and reabsorption (left to right in Figure 1b). These processes are strongly affected by repulsive Coulomb interactions among electrons and attractive interaction with the screening image charges created on the material surface.^18,19^ As a result, the charge distribution close to the surface exhibits strong spatial inhomogeneities on the nanometer/femtosecond scales, which remain largely unexplored in experiments^5,20^ despite their pivotal role in developing potential applications.

**Figure 1:**
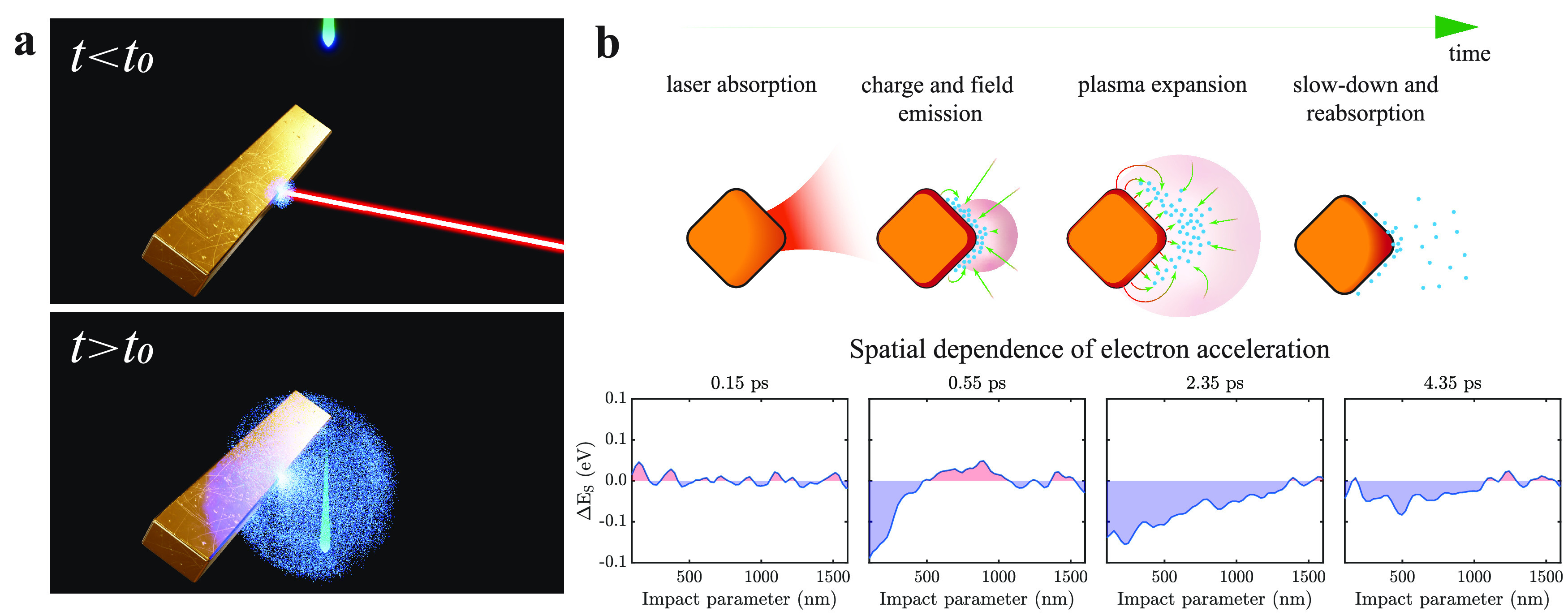
The CDEM technique and its application to image ultrafast nanoscale plasma dynamics. (a) Schematics of the studied phenomenon. A laser pulse (50 fs, 800 nm) generates a cloud of photoemitted electrons that is probed by an e-beam pulse (200 keV, 600 fs) with a tunable delay time relative to the laser pulse. (b) Differentiated stages (left to right) in the dynamics of the generated electron cloud (upper schemes) and its impact on the transmitted electrons (lower plots): initial laser irradiation; photoemission and THz field generation; explosive phase of rapid charge expansion; and charge density depletion via surface reabsorption. Lower plots show the average e-beam energy change Δ*E*_S_ as a function of e-beam–surface distance at selected delay times (upper labels).

Here, we introduce an approach to access the spatiotemporal dynamics of high-density photoemitted electron clouds: charge dynamics electron microscopy (CDEM), performed in an ultrafast transmission electron microscope (UTEM) in which 50 fs, 800 nm laser pulses are used to irradiate a metallic target and 600 fs electron-beam (e-beam) pulses are probing the dynamics of the emitted electrons (Figure 1a). The spectra of the electron pulses are then recorded as a function of e-beam spatial position and delay time relative to the laser pulses. The emission and subsequent dynamics of the charge cloud generate broadband low-frequency (THz) nonconservative electromagnetic fields, which produce a sizable overall acceleration of the transmitted e-beam. The dependence of the measured acceleration on e-beam position and delay time relative to the laser pulse reveals a wealth of information on the spatiotemporal dynamics of the electron cloud, as well as its interaction with the emitting material. The entire process involves strong dynamical screening of the exciting laser, ultrafast internal carrier dynamics and thermalization, thermionic and multiphoton photoemission, Coulomb interactions between free-space and image charges, electron–surface recollisions, the generation of low-frequency fields, and the interaction of the latter with the sampling e-beam. We supplement our experiments with a comprehensive microscopic theoretical analysis of these processes in excellent agreement with the measured data, allowing us to conclusively establish four well-differentiated stages of charge evolution, as illustrated in Figure 1b.

## Results and Discussion

**Figure 2:**
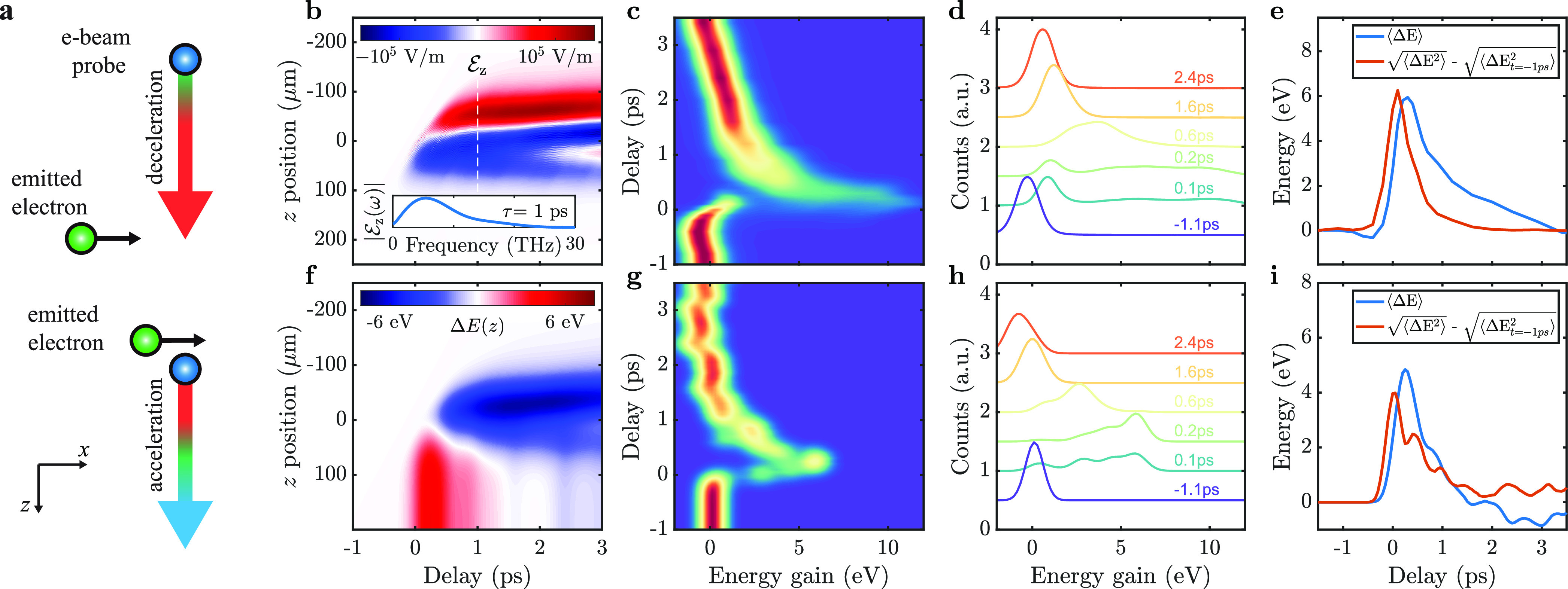
Ultrafast e-beam interactions in CDEM. (a) Sketches illustrating e-beam deceleration and acceleration stages, which result in an average net energy change, as well as spectral reshaping. (b) Simulated electric field  experienced by the e-beam as a function of delay and position along the trajectory (*z* = 0 corresponding to the e-beam leveled with the tip of the metal corner in Figure 1) for an e-beam–surface separation of 100 nm. The inset shows the Fourier-transformed electric-field amplitude at a delay of 1 ps (white dashed line), peaking at 5.4 THz. (c) Transmitted electron spectra as a function of laser–e-beam delay for 100 nm e-beam–surface separation. (d) Profiles extracted from panel c at selected delays (see labels). (e) Variation of the average e-beam energy and spectrum variance as a function of delay. (f) Calculated modification of e-beam energy Δ*E*(*z*) as a function of both electron position *z* and delay between optical and electron pulses as the electron experiences the effect of the electric field  in panel b. (g, h, i) Numerical simulations based on microscopic theory corresponding to the conditions in panels c, d, and e, respectively. The laser fluence is 189 mJ/cm^2^.

The main observable in our measurements is the spatial pattern of acceleration experienced by the energetic e-beam probe after passing next to or through the emitted electron cloud. As the latter evolves, it produces time-varying electromagnetic fields that comprise low-frequency components interacting with the e-beam (1–10 THz, see inset to Figure 2b and Figure S3 in SI). The acceleration of free electrons by THz fields has been previously investigated using, for example, point-projection electron microscopy.^21^ However, the CDEM technique performed in an UTEM represents a radical step forward in our ability to probe dense plasmas (10^14^ cm^–3^) of different geometries, sizes, and densities with a resolution in the nanometer/femtosecond range over a large field of view.

In our experiment, an electron cloud is photoemitted from a corner of a metal structure, expanding with drift kinetic energies of a fraction of an electronvolt. We find that the acceleration observed in the e-beam is predominantly caused by cloud charge motion along transverse directions, as schematically depicted in Figure 2a, while motion parallel to the e-beam contributes negligibly for the cloud velocities observed in our experiment. The dynamical character of the interaction is essential. In contrast, for quasi-static charge motion, as explored in deflectometry-based experiments,^20,22−27^ the deceleration and acceleration of the probe electron before and after transit are perfectly balanced and produce no net effect. Instead, for rapidly and noninertially evolving charges, the two contributions are unbalanced and result in a net energy transfer to the e-beam (Figure 2a).

A typical temporal evolution of the measured e-beam acceleration is presented in Figure 2c,d, which shows the measured change in the electron spectrum as a function of the delay time relative to the laser pulse for a fluence of 189 mJ/cm^2^. The temporal dynamics consists of strong electron acceleration and spectral broadening at short delays, followed by a slow reduction of the acceleration and, eventually, even deceleration (Figure 2c,d). Qualitatively, as inferred from the schematics in Figure 2a, a net acceleration is observed if there is a current flowing perpendicularly to the motion of the e-beam, as it introduces an imbalance in the average e-beam–plasma distance as the interaction occurs. In an intuitive picture, the observed acceleration is the result of the work done on the electron by the electric field  generated by the cloud electrons and image charges acting along the probe trajectory **r**_e_(*t*). The electron energy change is given by the time integral
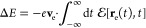
1where **v**_e_ is the e-beam velocity vector, taken to be approximately constant in the evaluation of eq 1. This expression, which represents the work done on a classical point-particle electron, can be rigorously derived from a quantum-mechanical treatment of the e-beam when the external THz field varies negligibly during the interaction time defined by τ_interaction_ ∼ *L*/*v*_e_, which is ∼1 ps for an effective interaction length *L* ∼ 200 μm (see Figure 2b) along the beam direction (see Methods).

To better understand the origin of the acceleration and estimate the effect of free and image charges, experimental geometry, and material properties, we compare the measured data with simulations based on a comprehensive account of the different microscopic physical processes involved in the generation and evolution of the electron cloud, as well as its interaction with the probing electron (see details of the theory in Methods and Supporting Information (SI)). Figure 2b shows the calculated electric-field component parallel to the e-beam as a function of both (i) the delay between laser and e-beam pulses and (ii) the position along the electron trajectory, while the inset shows its spectral decomposition at 1 ps delay. The resulting delay- and position-dependent variation of the e-beam energy is shown in Figure 2f, as obtained from eq 1 by setting the upper integration limit to a finite time corresponding to each electron-probe position (see also Figure S3 in SI). We observe that the electric field rapidly decays away from the metal and becomes negligible at distances >100 μm, beyond which the e-beam energy remains unchanged (i.e., at the value recorded in experiment). Accounting for the finite e-beam pulse duration, we also calculate the e-beam spectrum as a function of probe delay (Figure 2g,h), finding qualitative and quantitative agreement with the experiment.

To quantitatively capture both the amplitude of the acceleration and the observed time scales, two different emission processes need to be considered: thermionic, due to the increase in electron temperature, which stays elevated during a picosecond time scale; and three-photon photoemission, which occurs within the 50 fs duration of the laser pulse (see Methods for details). At each time step in the simulation, the force exerted on every individual electron by the remaining electrons and their associated induced surface image charges is evaluated, and its position and velocity are evolved accordingly (see Figure S2 in SI for details on the plasma charge dynamics). Partial electron absorption upon recollision with the metal surface is also accounted for. The net energy variation of a probing electron after traversing the plasma is then calculated from eq 1, with the electric field obtained by summing the contributions from all emitted cloud electrons and their associated image charges, including the effect of surface geometry and retardation, and further averaging over the electron wavepacket density profile (see SI). Our simulations reveal that the contribution of the interactions with the electron cloud and the image charges produce two components of similar amplitude but with opposite signs (see Figure S1c in SI). However, image charges are constrained to the material surface, so their contribution is weaker than that of free-space charges. Effectively, the e-beam probe sees an effective dipolar field, with the dipole oriented nearly transversely with respect to the beam direction.

As shown in Figure 2e, our CDEM measurements unveil two main time scales: (i) fast plasma creation by thermionic and photoemission processes occurring faster than the electron pulse duration (fwhm ≃ 600 fs); and (ii) plasma dynamics driven by space and image charges, which manifests as a gradual relaxation of the electron energy shift Δ*E* over 1–2 ps. During the former, in addition to the net acceleration, we observe a substantial broadening of the electron spectrum, which we quantify by computing the second moment . This experimental broadening, which is also captured in our simulations, exhibits a maximum at the delay for which we encounter the largest variation of the average acceleration (Figure 2e,i), so that peak broadening and peak acceleration are mutually delayed by ∼200 fs.

The presence of free-space and image charges drastically affects the expansion and evolution of the electron cloud.^18,28,29^ For example, charge expansion is close to ballistic at low fluences, when emitted charge densities are small. In contrast, when the cloud reaches large densities, newly emitted electrons are trapped closer to the surface due to the strong Coulomb repulsion by previously emitted electrons,^18,19^ causing the number of electrons that permanently escape the photoexcitation region to be drastically reduced down to only a fraction ∼10^–6^–10^–8^ of the total emission.^18^ Those that acquire sufficient velocity to escape the photoexcitation region can be investigated by electron detectors and imaged with electron-deflection-based techniques,^20,22−27^ while in this work we provide insight into the previously inaccessible high-density electron cloud that is eventually reabsorbed during the first few picoseconds after emission.

**Figure 3:**
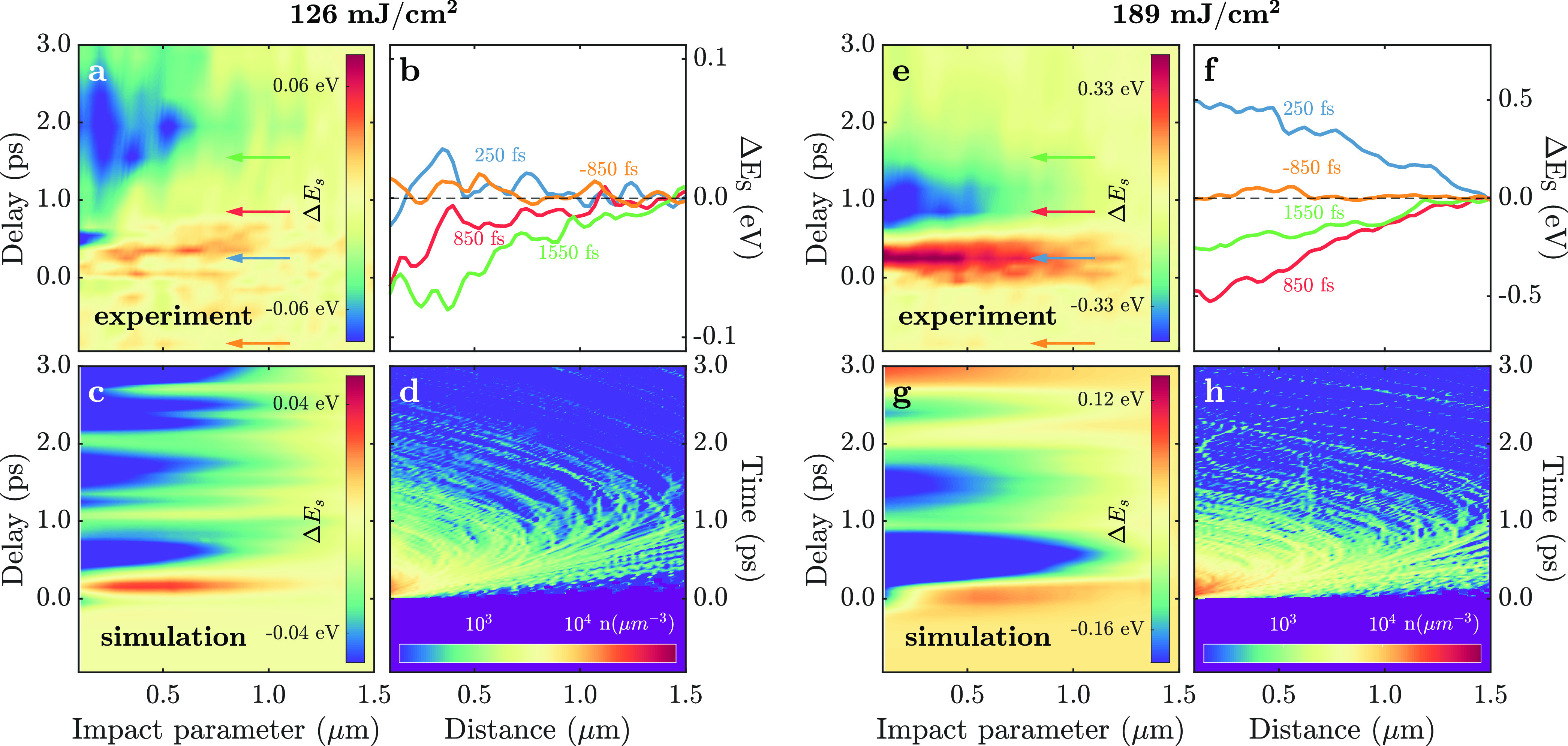
Temporal evolution of the spatial variation of the average electron acceleration. (a) Experimentally measured temporal evolution of the spatial variation of electron acceleration Δ*E*_S_ under excitation with 126 mJ/cm^2^ laser pulse fluence. (b) Selected spatial profiles of Δ*E*_S_ from panel a. (c) Theoretically calculated Δ*E*_S_ with plasma evolution simulated under the same conditions as in panel a. (d) Simulated density of plasma electrons as a function of time and separation from the metallic surface. Panels e, f, g, and h are the same as panels a, b, c, and d, respectively, but for 189 mJ/cm^2^ laser pulse fluence.

The spatial extension of the expanding charged plasma, its initial velocity, and the deceleration due to interaction with the image charges are all pivotal elements of information that can be extracted by studying the spatial variation of the e-beam probe acceleration in CDEM. In Figure 3, we present the spatial variation of the e-beam energy change as a function of delay time and beam position: Δ*E*_S_(*t*, *d*) = ⟨*E*⟩(*t*, *d*) – ⟨*E*⟩(*t*, *d*_max_), referred to the e-beam–target separation for the maximum explored impact parameter *d*_max_ = 1.5 μm. This allows us to precisely follow the spatial dynamics developing over the average acceleration. Experimental results for 126 and 189 mJ/cm^2^ laser pulse fluence are shown in Figure 3a,e, respectively. Close to the metal surface, Δ*E*_S_ is positive at early delay times, while it becomes negative at later delay times. This negative feature is characterized by faster rise and decay times when irradiating with a larger fluence (see selected profiles in Figure 3b,f).

Figure 3c,g shows the corresponding numerical simulations for Δ*E*_S_, qualitatively reproducing the experimental features, including their time scales and variation with fluence. Some quantitative differences are observed, which we attribute to the limited precision of the theoretical results (note that Δ*E*_S_/Δ*E*_max_ ∼ 1%) associated with constraints on the minimum time step and spatial discretization grid in the simulations (see more details in SI). However, the order of magnitude of the observed effects is successfully reproduced.

A comparison with the evolution of the emitted electron density (Figure 3d,h) allows us to gain further insight into the relationship between the observed behavior of Δ*E*_S_ and the charge dynamics, understand the origin of the spatial inhomogeneities in the acceleration, and hence derive important quantities such as the initial plasma expansion velocity. While Figure 3d,h shows the evolution of electron density as a function of distance from the surface, we probe experimentally the integral along the electron trajectory at a given impact parameter. At short delay times, the emitted charge cloud is localized close to the surface, and the spatial variation of the observed acceleration in Figure 3a,e is reminiscent of the power-law dependence of the near-field THz component, in accordance with eqs S12 and S13 in SI, taking into account the extended three-dimensional shape of the emitted charge. This regime is not directly observed in Figure 3d,h. As the charge cloud expands, at distances substantially outside the cloud, the acceleration displays a similar decaying component. However, as the probe electron passes through the cloud, the effect of partial screening causes a reduction in the electron beam acceleration, as manifested in the negative Δ*E*_S_ region in Figure 3a,e, whose onset permits us to experimentally determine the initial charge expansion velocity as ∼1.2 nm/fs.

Due to the interaction with image charges, most of the emitted electrons slow down in the immediate vicinity of the surface and are eventually reabsorbed. This is confirmed upon inspection of individual particle trajectories in our theory (Figure 3d,h), which bend to the surface and eventually collide with it within a few hundred femtoseconds, while those that acquire higher speed in the initial stage are able to escape the surface-neighboring region. Electrons that are colliding with the surface do not observably influence the e-beam probe spectrum because of their reduced speed and the canceling fields originating in proximal positive (image) and negative (electron) charges.

Expansion and reabsorption of the electron cloud result in a reduction of the cloud density (see Figure SI2a), which leads in turn to a gradual depletion of the negative Δ*E*_S_ region close to the sample surface on a 1–2 ps time scale (see Figure 3a,c,e,g).

### Future Directions

**Figure 4:**
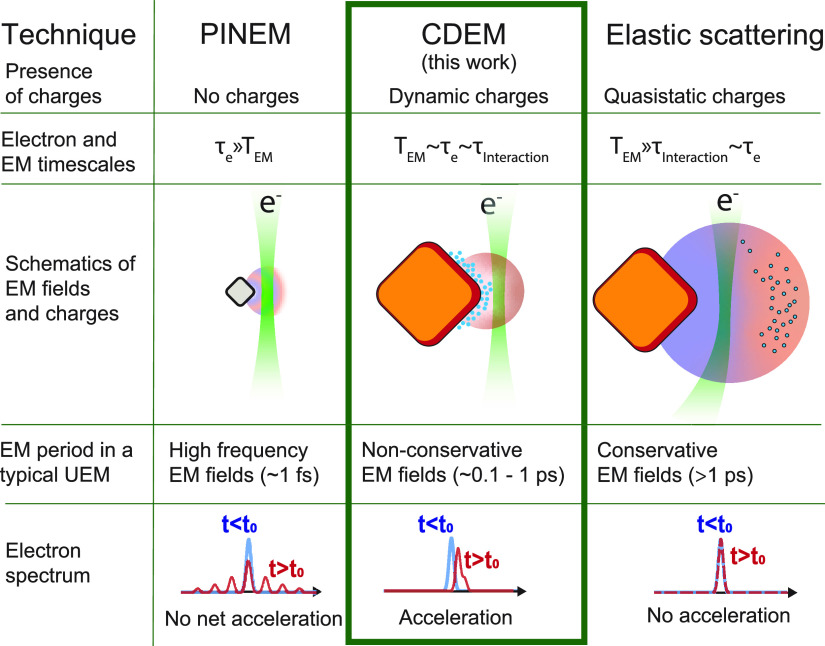
Comparison between different ultrafast electron microscopy techniques and the corresponding interactions between the probe electrons and electromagnetic near fields. The table compares relevant parameters and the main differences between the techniques, relating to time scales and experimental observables. EM, electromagnetic; UEM, ultrafast electron microscope; CDEM, charge dynamics electron microscopy; PINEM, photon-induced near-field electron microscopy.

In perspective, CDEM covers a previously unexplored regime of ultrafast interaction between e-beams and near fields, as emphasized in Figure 4, which compares CDEM both to photon-induced near-field electron microscopy (PINEM)^30−32^ and to electron microscopy methods based on elastic electron–field interactions.^20,22−27^ The latter (Figure 4, right column) involves an optical cycle of the electromagnetic field *T*_EM_ that is large compared with both τ_interaction_ and the electron pulse duration τ_e_. This regime includes Lorentz transmission electron microscopy, electron holography, deflectometry, and shadowgraphy, which are sensitive to slow quasistatic conservative electric fields.^23,33^ On the opposite extreme, PINEM (Figure 4, left column) capitalizes on the effect of rapidly oscillating optical fields (*T*_EM_ ≪ τ_e_), which show up as inelastic peaks in the electron spectrum at multiples of the photon energy, usually configuring a symmetric spectrum (for nearly monochromatic illumination) with respect to the elastic peak due to the stimulated nature of the process and the large occupation number of the involved laser-driven optical modes. Under exposure to monochromatic fields, the net e-beam energy change in PINEM is zero, just like in elastic diffraction techniques. This is one key aspect by which CDEM deviates from other techniques: the electron spectrum is asymmetric, producing a sizable e-beam energy change. Indeed, the intermediate regime in which the interaction, electron-pulse, and optical-cycle times are commensurate (Figure 4, central column) is where CDEM belongs: a natural domain to extract spatiotemporal information on the probed fields and associated sources. A unified, rigorous quantum-mechanical formalism can simultaneously capture all three regimes with a relatively simple theory (see Methods), under the approximation that the kinetic energy of the incident probe electron largely exceeds the energy change due to the interaction, as is the case here. In such a scenario, the incident wave function is multiplied by a factor involving the exponential of an action (the integrated field along the probe trajectory), which becomes an energy comb for monochromatic fields (i.e., the PINEM limit); in contrast, the same factor reduces to the energy shift given by eq 1 in the classical limit (see Methods).

In contrast to PINEM and elastic scattering, the CDEM approach allows us to follow the formation and evolution of dense plasma with nanometer/femtosecond space/time resolution. From a technological viewpoint, access to spatially resolved information offers the possibility to develop customized nanostructures that can be optimized to operate on ultrafast time scales.^8^ Also, from a material science perspective, CDEM enables the investigation of image charge dynamics and screening time scales in out-of-equilibrium nanostructured materials, allowing us to map spatial inhomogeneities such as the formation of domains following a phase transition.

## Conclusions

Through the insight gathered from CDEM on free-space electron clouds, combined with a predictive degree of theoretical modeling, we introduce a powerful tool for the quantitative optimization of electron sources operating under extreme space-charge conditions. This has potential application in nanopatterned radiofrequency-gun electron emitters, where 100 nm sized features have been demonstrated to produce a 100-fold electron yield enhancement.^34^ Similarly, periodic arrays of electron-plasma emitters can drastically improve the emission efficiency on the femtosecond scale by operating in a high-plasma-density regime exceeding critical values by orders of magnitude.^17^ CDEM is an ideal tool to diagnose such supercritical plasma, providing nanometer/femtosecond space/time-resolved imaging to optimize geometrical and compositional parameters. Similar benefits are expected in the development of plasma-based high-efficiency X-ray sources, nanoelectronic devices, and nuclear or astrophysics-in-a-lab experiments.^35^

The intense nanoscale THz fields produced by the diagnosed plasma hold strong potential for use in the spatial, angular, and spectral compression of e-beams, enabling finer spatiotemporal control with respect to traditional THz-based approaches.^36,37^ CDEM could thus be applied to manipulate the wave function of free electrons in ways that existing techniques such as PINEM cannot. In addition, the photon statistics of the THz field associated with the out-of-equilibrium plasma remains as a fundamental question^38^ that cannot be addressed with conventional quantum-optics techniques because of the limited speed and sensitivity of available THz photodetectors.^39−41^ CDEM is thus offering a viable approach to characterize the statistics of near-field photons at low frequencies.

## Methods

### Sample Preparation and UTEM Experiments

For the experiments reported, we used a copper 100 Mesh PELCO grid. The grid was tilted by 45° with respect to the *z* direction (parallel to the TEM column axis) in order to expose the corner of a rectangular copper rod with a cross section of ∼50 × 25 μm^2^ (see Figure S1 in SI). The edge of the rod corner exhibited a radius of curvature of 4 μm, as estimated from SEM micrographs. The sample was positioned such that only one of the edges of the rectangular rod was illuminated by the laser pulse.

To generate the charged plasma, we irradiated the copper rod with near-infrared laser pulses of 1.55 eV central photon energy (800 nm) and 50 fs temporal duration at a repetition rate of 100 kHz, which corresponds to ≃2.5 TW/cm^2^. In the reported experiments, light polarization was vertical (i.e., along the propagation direction of the probe electron). Light entered the microscope through the zero-angle port and was focused under normal incidence on the copper rod via an external plano-convex lens. In such a geometry, the light beam was also perpendicular with respect to the electron propagation direction.

The dynamics of the photoemitted electrons was then probed by means of electron pulses with a temporal duration of about 600 fs and with a controlled delay between electron and laser pulses. All the experiments were performed in a modified JEOL 2100 TEM microscope at an acceleration voltage of 200 kV.^42,43^ The probe electrons were generated by illuminating a LaB_6_ cathode with third-harmonics UV light at 4.65 eV photon energy.

Our transmission electron microscope was equipped with EELS capabilities coupled to real-space imaging. Energy-resolved spectra were recorded using a Gatan-Imaging-Filter (GIF) camera operated with a 0.05 eV-per-channel dispersion setting and typical exposure times of the CCD sensor from 30 to 60 s. For the acquisition of space-energy maps (see Figure 3), special care was devoted to sample alignment. The copper rod was adjusted to be parallel to the energy dispersion direction and placed at the edge of the spectrometer entrance aperture.

The acquired position-dependent spectra were analyzed as a function of delay between the laser and electron pulses, with the time zero being determined as the peak of PINEM signal observed within 100 nm close to the sample surface at relatively low fluence (≃50 mJ/cm^2^). Camera noise and signal from cosmic events were reduced by applying median filtering. Distortions of the spectrometer were corrected by aligning the spectrum according to the negative delay energy-space spectrographs (−2 ps). The first and second moments of the spectrum were calculated in a reduced energy window, which was taken 10 eV larger than the region in which the electron signal was above 10% of the peak value (i.e., the maximum value among all delays and positions measured for a given fluence). This procedure helped to reduce contributions from the CCD background noise.

Regarding sample stability, special care was taken to ensure experimentally reproducible results and a controlled environment. Standard TEM grids from the same batch were used for all the experiments. The oxide layer was removed from the surface by washing the grids in acetic acid for approximately 5 min. Among other reasons, relatively fine-pitch grids were selected to avoid resonant vibrations due to a large periodic thermal load. In experiments, the smearing of the sample edge did not exceed the resolution of ≃50 nm defined by the magnification settings and aberrations in the photoelectron mode of TEM operation. At the highest measured fluence, we observed a degradation of the signal of the order of ≃10% of the peak acceleration over 2 h of experimental time. We made sure to expose a fresh part of the sample to laser illumination at least every 60 min. Sample edge images were realigned for each measurement during data analysis. At fluences above 500 mJ/cm^2^, we observed ablation of the sample on a time scale of several minutes.

### Classical Limit for the Energy Loss Experienced by a Free Electron Traversing an Optical Field

We derive a classical limit for the interaction between a collimated free electron and a classical electromagnetic field starting from a quantum-mechanical expression that bears general validity in the nonrecoil approximation.

Under the experimental conditions, the free electron probe has a small energy spread relative to its average kinetic energy both before and after the interaction. We can therefore adopt the nonrecoil approximation^44^ and introduce the interaction with a classical field through the Hamiltonian (*ev*/*c*)*A*_*z*_, where *v* is the electron velocity and *A*_*z*_ is the vector potential component along the beam direction *z* in the Coulomb gauge, for which the scalar potential vanishes within the vacuum space traversed by the electron. We further consider a finite interaction region, in which *v* is assumed to remain constant, such that the wave function depends on the longitudinal coordinate *z* and time *t* only through *z* – *vt*. Under these conditions, starting from an incident electron wave function ψ^0^(*z*, *t*), the postinteraction wave function reduces to^32,44,45^
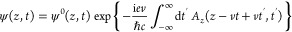
2where an implicit dependence on transverse coordinates (*x*, *y*) is understood.

The evaluation of eq 2 for either monochromatic fields or optical pulses of short duration compared with the optical period reduces the exponential factor to a well-known sum over energy sidebands that accurately describes experimentally observed PINEM spectra.^45^ In contrast, in the present work, the electron is exposed to external fields comprising components whose optical cycles are long compared to the interaction time *L*/*v* (see main text). It is then pertinent to Taylor-expand the slowly varying vector potential *A*_*z*_(*z* – *vt* + *z*′, *t*′) around small values of *z* – *vt*, assuming the centroid of the electron wavepacket to follow the trajectory *z* = *vt*. The independent term in this expansion contributes with an overall phase φ = −(*ev*/ℏ*c*)∫_–*∞*_^*∞*^d*tA*_*z*_(*vt*, *t*) that does not affect the transmitted electron spectrum. Retaining only the linear term in *z* – *vt*, eq 2 reduces to
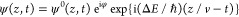
3where
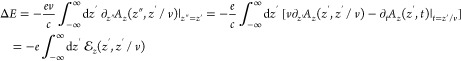
4represents the energy change experienced by a classical point electron moving along the noted trajectory. In the derivation of this expression, the first term in the second line cancels upon integration by parts for a field of finite extension along the electron trajectory (i.e., localized at the interaction region), and we have identified 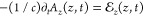
with the electric field component along the beam direction to obtain the third line. In summary, the wave function in eq 3 is the incident one multiplied by an irrelevant phase factor e^iφ^ as well as by a plane wave e^i(Δ*E*/*ℏ*)(*z*/*v*–*t*)^ representing a rigid shift in energy by Δ*E* (and a corresponding change in momentum by Δ*E*/*v* within the nonrecoil approximation). From eq 4, we then recover eq 1 by setting *z*′ = *vt*. Corrections of higher-order terms in the aforementioned Taylor expansion may become relevant for electron wavepacket durations similar to or larger than either the optical cycle or the temporal extension of the external field.

### Numerical Simulations

In this section, we describe the main aspects of the theoretical model employed to simulate the experimental results presented in this work. Additional details can be found in SI.

First, we model the temperature dynamics *T*(*t*, *s*) in the inner surface of the copper bar as a function of time *t* and surface position *s* using the two-temperature model (see Figure S1 in SI). The pump illumination is introduced through the near-field distribution calculated in the inner surface through the boundary-element method.^51^

We then model electron emission as a function of local temperature *T* from two different channels: thermionic emission, due to the heightened temperature of the surface electrons, which extends over a few picoseconds and we evaluate using a surface-barrier model; and three-photon photoemission, resulting from the absorption of three photons by one electron during the duration of the pumping <100 fs, calculated using the Fowler–Dubridge model (see SI for details). For the latter, we have used a likelihood of emission parameter *a*_3_ ∼ 0.5 × 10^–35^ cm^6^/A^3^ that is an order of magnitude lower than previously reported estimates in copper,^46,47^ which we attribute primarily to a saturation effect due to the high pump laser fluences used in this work^48^ (see SI for further discussion).

Combining these results, we simulate the emission of photoelectrons at each instant of time and surface position, which gives rise to a density of photoemitted electrons ρ_e_(**R**, *t*) as a function of spatial position **R** and time *t*. The evolution of the plasma density is then simulated by discretizing time and considering, at each time step, the force acting on each of the photoemitted electrons by all the remaining ones, as well as the interaction with the copper bar. The latter is introduced by rigorously accounting for the accumulation of positive image charges at the copper surface due to the presence of the negatively charged electrons in its vicinity. The position and velocity of each electron is then evolved according to the net force exerted on it. The eventual collision of the photoemitted electrons with the surface gives rise to partial reabsorption according to the barrier model, as well as specular reflection of the nonabsorbed electrons. This procedure allows us to determine ρ_e_(**R**, *t*) for the full duration of the simulation (see Figure S2 in SI).

Finally, we calculate the energy variation by a probe electron passing with velocity **v**_e_ at a distance *b* from the copper bar along a trajectory **r**_e_(*t*) = **r**_0_ + **v**_e_(*t* − τ), where **r**_0_ is the nearest point to the copper bar and τ is the delay of the probe electron with respect to the laser pump. At each time *t*, we calculate the electric field  at the probe electron position **r**_e_(*t*) generated by all of the plasma electrons and their corresponding surface charges, taking into account retardation effects and averaging over the time duration of the electron wavepacket (see SI for additional details). The net energy variation by the probe electron, which is a function of *b* and τ, is finally obtained by using eq 1.

## Supporting Information Available

Movie of time evolution of the electron energy variation (MP4)
Methods for numerical simulations of surface temperature dynamics, electronic emission, charge dynamics, and energy variation of the probe electron, numerical simulation details, plasma charge dynamics, energy variation of the probe electron, and ultrafast e-beam interactions in CDEM under 126 mJ/cm^2^ laser pulse (PDF)

## References

[ref1] Vanacore G. M.; Fitzpatrick A. W.; Zewail A. H. Four-Dimensional Electron Microscopy: Ultrafast Imaging, Diffraction and Spectroscopy in Materials Science and Biology. Nano Today 2016, 11, 228–249. 10.1016/j.nantod.2016.04.009.

[ref2] Sobota J. A.; He Y.; Shen Z. X. Angle-Resolved Photoemission Studies of Quantum Materials. Rev. Mod. Phys. 2021, 93, 025006 10.1103/RevModPhys.93.025006.

[ref3] Maiuri M.; Garavelli M.; Cerullo G. Ultrafast Spectroscopy: State of the Art and Open Challenges. J. Am. Chem. Soc. 2020, 142, 3–15. 10.1021/jacs.9b10533. 31800225

[ref4] He Z.-H.; Thomas A. G. R.; Beaurepaire B.; Nees J. A.; Hou B.; Malka V.; Krushelnick K.; Faure J. Electron Diffraction Using Ultrafast Electron Bunches from a Laser-Wakefield Accelerator at kHz Repetition Rate. Appl. Phys. Lett. 2013, 102, 064104 10.1063/1.4792057.

[ref5] Musumeci P.; Moody J. T.; Scoby C. M.; Gutierrez M. S.; Westfall M.; Li R. K. Capturing Ultrafast Structural Evolutions with a Single Pulse of MeV Electrons: Radio Frequency Streak Camera Based Electron Diffraction. J. Appl. Phys. 2010, 108, 114513 10.1063/1.3520283.

[ref6] Lange S. L.; Noori N. K.; Kristensen T. M. B.; Steenberg K.; Jepsen P. U. Ultrafast THz-Driven Electron Emission from Metal Metasurfaces. J. Appl. Phys. 2020, 128, 070901 10.1063/1.5142590.

[ref7] Huang W. R.; Fallahi A.; Wu X.; Cankaya H.; Calendron A.-L.; Ravi K.; Zhang D.; Nanni E. A.; Hong K.-H.; Kärtner F. X. Terahertz-Driven, All-Optical Electron Gun. Optica 2016, 3, 1209 10.1364/OPTICA.3.001209.

[ref8] Samizadeh Nikoo M.; Jafari A.; Perera N.; Zhu M.; Santoruvo G.; Matioli E. Nanoplasma-Enabled Picosecond Switches for Ultrafast Electronics. Nature 2020, 579, 534–539. 10.1038/s41586-020-2118-y. 32214267

[ref9] Miaja-Avila L.; O’Neil G. C.; Joe Y. I.; Alpert B. K.; Damrauer N. H.; Doriese W. B.; Fatur S. M.; Fowler J. W.; Hilton G. C.; Jimenez R.; Reintsema C. D.; Schmidt D. R.; Silverman K. L.; Swetz D. S.; Tatsuno H.; Ullom J. N. Ultrafast Time-Resolved Hard X-Ray Emission Spectroscopy on a Tabletop. Physical Review X 2016, 6, 031047 10.1103/PhysRevX.6.031047.

[ref10] Fullagar W.; Harbst M.; Canton S.; Uhlig J.; Walczak M.; Wahlström C.-G.; Sundström V. A Broadband Laser Plasma X-Ray Source for Application in Ultrafast Chemical Structure Dynamics. Rev. Sci. Instrum. 2007, 78, 115105 10.1063/1.2813340. 18052502

[ref11] Bargheer M.; Zhavoronkov N.; Gritsai Y.; Woo J. C.; Kim D. S.; Woerner M.; Elsaesser T. Coherent Atomic Motions in a Nanostructure Studied by Femtosecond X-Ray Diffraction. Science 2004, 306, 1771–1773. 10.1126/science.1104739. 15576618

[ref12] Sokolowski-Tinten K.; Blome C.; Dietrich C.; Tarasevitch A.; Horn von Hoegen M.; von der Linde D.; Cavalleri A.; Squier J.; Kammler M. Femtosecond X-Ray Measurement of Ultrafast Melting and Large Acoustic Transients. Phys. Rev. Lett. 2001, 87, 225701–225701–4. 10.1103/PhysRevLett.87.225701. 11736408

[ref13] Higashiguchi T.; Dojyo N.; Hamada M.; Sasaki W.; Kubodera S. Low-debris, Efficient Laser-Produced Plasma Extreme Ultraviolet Source by Use of a Regenerative Liquid Microjet Target Containing Tin Dioxide (SnO2) Nanoparticles. Appl. Phys. Lett. 2006, 88, 201503 10.1063/1.2206131.

[ref14] Nie Z.; Pai C. H.; Hua J.; Zhang C.; Wu Y.; Wan Y.; Li F.; Zhang J.; Cheng Z.; Su Q.; Liu S.; Ma Y.; Ning X.; He Y.; Lu W.; Chu H. H.; Wang J.; Mori W. B.; Joshi C. Relativistic Single-Cycle Tunable Infrared Pulses Generated from a Tailored Plasma Density Structure. Nat. Photonics 2018, 12, 489–494. 10.1038/s41566-018-0190-8.

[ref15] Wu Y.; Gunst J.; Keitel C. H.; Pálffy A. Tailoring Laser-Generated Plasmas for Efficient Nuclear Excitation by Electron Capture. Phys. Rev. Lett. 2018, 120, 052504 10.1103/PhysRevLett.120.052504. 29481161

[ref16] Gunst J.; Wu Y.; Keitel C. H.; Pálffy A. Nuclear Excitation by Electron Capture in Optical-Laser-Generated Plasmas. Phys. Rev. E 2018, 97, 063205 10.1103/PhysRevE.97.063205. 30011546

[ref17] Samsonova Z.; Höfer S.; Kaymak V.; Ališauskas S.; Shumakova V.; Pugžlys A.; Baltuška A.; Siefke T.; Kroker S.; Pukhov A.; Rosmej O.; Uschmann I.; Spielmann C.; Kartashov D. Relativistic Interaction of Long-Wavelength Ultrashort Laser Pulses with Nanowires. Physical Review X 2019, 9, 021029 10.1103/PhysRevX.9.021029.

[ref18] Wendelen W.; Autrique D.; Bogaerts A. Space Charge Limited Electron Emission from a Cu Surface under Ultrashort Pulsed Laser Irradiation. Appl. Phys. Lett. 2010, 96, 051121 10.1063/1.3292581.

[ref19] Tao S.; Wu B. Early-Stage Effects of Residual Charges in a Metal Target on Emitted Electrons Induced by Femtosecond Laser–Metal Interactions. Physics Letters A 2017, 381, 404–407. 10.1016/j.physleta.2016.10.060.

[ref20] Schäfer S.; Liang W.; Zewail A. H. Structural Dynamics and Transient Electric-Field Effects in Ultrafast Electron Diffraction from Surfaces. Chem. Phys. Lett. 2010, 493, 11–18. 10.1016/j.cplett.2010.04.049.

[ref21] Hergert G.; Wöste A.; Vogelsang J.; Quenzel T.; Wang D.; Gross P.; Lienau C. Probing Transient Localized Electromagnetic Fields Using Low-Energy Point-Projection Electron Microscopy. ACS Photonics 2021, 8, 2573–2580. 10.1021/acsphotonics.1c00775.

[ref22] Zandi O.; Sykes A. E.; Cornelius R. D.; Alcorn F. M.; Zerbe B. S.; Duxbury P. M.; Reed B. W.; van der Veen R. M. Transient Lensing from a Photoemitted Electron Gas Imaged by Ultrafast Electron Microscopy. Nat. Commun. 2020, 11, 3001 10.1038/s41467-020-16746-z. 32532996 PMC7293293

[ref23] Sun S.; Sun X.; Bartles D.; Wozniak E.; Williams J.; Zhang P.; Ruan C. Y. Direct Imaging of Plasma Waves using Ultrafast Electron Microscopy. Structural Dynamics 2020, 7, 064301 10.1063/4.0000044. 33415182 PMC7772000

[ref24] Scoby C. M.; Li R. K.; Musumeci P. Effect of an Ultrafast Laser Induced Plasma on a Relativistic Electron Beam to Determine Temporal Overlap in Pump-Probe Experiments. Ultramicroscopy 2013, 127, 14–18. 10.1016/j.ultramic.2012.07.015. 22951263

[ref25] Hebeisen C. T.; Sciaini G.; Harb M.; Ernstorfer R.; Kruglik S. G.; Miller R. J. D. Direct Visualization of Charge Distributions during Femtosecond Laser Ablation of a Si (100) Surface. Phys. Rev. B 2008, 78, 081403 10.1103/PhysRevB.78.081403.

[ref26] Li J.; Wang X.; Chen Z.; Clinite R.; Mao S. S.; Zhu P.; Sheng Z.; Zhang J.; Cao J. Ultrafast Electron Beam Imaging of Femtosecond Laser-Induced Plasma Dynamics. J. Appl. Phys. 2010, 107, 083305 10.1063/1.3380846.

[ref27] Raman R. K.; Tao Z.; Han T. R.; Ruan C. Y. Ultrafast Imaging of Photoelectron Packets Generated from Graphite Surface. Appl. Phys. Lett. 2009, 95, 181108 10.1063/1.3259779.

[ref28] Riffe D. M.; More R. M.; Wang X. Y.; Downer M. C.; Fisher D. L.; Tajima T.; Erskine J. L. Femtosecond Thermionic Emission from Metals in the Space-Charge-Limited Regime. Journal of the Optical Society of America B 1993, 10, 1424 10.1364/JOSAB.10.001424.

[ref29] Wendelen W.; Mueller B.; Autrique D.; Rethfeld B.; Bogaerts A. Space Charge Corrected Electron Emission from an Aluminum Surface under Non-Equilibrium Conditions. J. Appl. Phys. 2012, 111, 113110 10.1063/1.4729071.

[ref30] Barwick B.; Flannigan D. J.; Zewail A. H. Photon-Induced Near-Field Electron Microscopy. Nature 2009, 462, 902–906. 10.1038/nature08662. 20016598

[ref31] García de Abajo F. J.; Asenjo-Garcia A.; Kociak M. Multiphoton Absorption and Emission by Interaction of Swift Electrons with Evanescent Light Fields. Nano Lett. 2010, 10, 1859–1863. 10.1021/nl100613s. 20415459

[ref32] Park S. T.; Lin M.; Zewail A. H. Photon-Induced Near-Field Electron Microscopy (PINEM): Theoretical and Experimental. New J. Phys. 2010, 12, 123028 10.1088/1367-2630/12/12/123028.

[ref33] Centurion M.; Reckenthaeler P.; Trushin S. A.; Krausz F.; Fill E. E. Picosecond Electron Deflectometry of Optical-Field Ionized Plasmas. Nat. Photonics 2008, 2, 315–318. 10.1038/nphoton.2008.77.

[ref34] Li R. K.; To H.; Andonian G.; Feng J.; Polyakov A.; Scoby C. M.; Thompson K.; Wan W.; Padmore H. A.; Musumeci P. Surface-Plasmon Resonance-Enhanced Multiphoton Emission of High-Brightness Electron Beams from a Nanostructured Copper Cathode. Phys. Rev. Lett. 2013, 110, 074801 10.1103/PhysRevLett.110.074801. 25166375

[ref35] Zhang P.; Ang Y. S.; Garner A. L.; Valfells Á.; Luginsland J. W.; Ang L. K. Space-Charge Limited Current in Nanodiodes: Ballistic, Collisional, and Dynamical Effects. J. Appl. Phys. 2021, 129, 100902 10.1063/5.0042355.

[ref36] Ehberger D.; Mohler K. J.; Vasileiadis T.; Ernstorfer R.; Waldecker L.; Baum P. Terahertz Compression of Electron Pulses at a Planar Mirror Membrane. Physical Review Applied 2019, 11, 024034 10.1103/PhysRevApplied.11.024034.

[ref37] Kealhofer C.; Schneider W.; Ehberger D.; Ryabov A.; Krausz F.; Baum P. All-Optical Control and Metrology of Electron Pulses. Science 2016, 352, 429–433. 10.1126/science.aae0003. 27102476

[ref38] Dahan R.; Gorlach A.; Haeusler U.; Karnieli A.; Eyal O.; Yousefi P.; Segev M.; Arie A.; Eisenstein G.; Hommelhoff P.; Kaminer I. Imprinting the Quantum Statistics of Photons on Free Electrons. Science 2021, 373, eabj7128 10.1126/science.abj7128. 34446445

[ref39] Kitaeva G. K.; Yakunin P. V.; Kornienko V. V.; Penin A. N. Absolute Brightness Measurements in the Terahertz Frequency Range using Vacuum and Thermal Fluctuations as References. Appl. Phys. B: Laser Opt. 2014, 116, 929–937. 10.1007/s00340-014-5779-0.

[ref40] Kutas M.; Haase B.; Bickert P.; Riexinger F.; Molter D.; von Freymann G. Terahertz Quantum Sensing. Science Advances 2020, 6, eaaz8065 10.1126/sciadv.aaz8065. 32201731 PMC7069706

[ref41] Prudkovskii P.; Leontyev A.; Kuznetsov K.; Kitaeva G. Towards Measuring Terahertz Photon Statistics by a Superconducting Bolometer. Sensors 2021, 21, 4964 10.3390/s21154964. 34372199 PMC8347001

[ref42] Piazza L.; Masiel D.; LaGrange T.; Reed B.; Barwick B.; Carbone F. Design and Implementation of a fs-Resolved Transmission Electron Microscope Based on Thermionic Gun Technology. Chem. Phys. 2013, 423, 79–84. 10.1016/j.chemphys.2013.06.026.

[ref43] Piazza L.; Cottet M.; Carbone F.; Masiel D.; LaGrange T. Principles and Implementation of an Ultrafast Transmission Electron Microscope. Microscopy and Microanalysis 2012, 18, 600–601. 10.1017/S1431927612004850.

[ref44] García de Abajo F. J.; Di Giulio V. Optical Excitations with Electron Beams: Challenges and Opportunities. ACS Photonics 2021, 8, 945–974. 10.1021/acsphotonics.0c01950. 35356759 PMC8939335

[ref45] Vanacore G. M.; Madan I.; Berruto G.; Wang K.; Pomarico E.; Lamb R. J.; McGrouther D.; Kaminer I.; Barwick B.; García de Abajo F. J.; Carbone F. Attosecond Coherent Control of Free-Electron Wave Functions using Semi-Infinite Light Fields. Nat. Commun. 2018, 9, 2694 10.1038/s41467-018-05021-x. 30002367 PMC6043599

[ref51] García de Abajo F. J.; Howie A. Retarded field calculation of electron energy loss in inhomogeneous dielectrics. Phys. Rev. B 2002, 65, 115418 10.1103/PhysRevB.65.115418.

[ref46] Musumeci P.; Cultrera L.; Ferrario M.; Filippetto D.; Gatti G.; Gutierrez M.; Moody J.; Moore N.; Rosenzweig J.; Scoby C.; et al. Multiphoton Photoemission from a Copper Cathode Illuminated by Ultrashort Laser Pulses in an RF Photoinjector. Physical review letters 2010, 104, 084801 10.1103/PhysRevLett.104.084801. 20366937

[ref47] Tsang T.; Srinivasan-Rao T.; Fischer J. Surface-Plasmon Field-Enhanced Multiphoton Photoelectric Emission from Metal Films. Phys. Rev. B 1991, 43, 8870 10.1103/PhysRevB.43.8870. 9996555

[ref48] Fujimoto J.; Liu J.; Ippen E.; Bloembergen N. Femtosecond Laser Interaction with Metallic Tungsten and Nonequilibrium Electron and Lattice Temperatures. Phys. Rev. Lett. 1984, 53, 1837 10.1103/PhysRevLett.53.1837.

[ref49] Yannai M.; Dahan R.; Gorlach A.; Rivera N.; Wang K.; Vanacore G. M.; Carbone F.; García de Abajo F. J.; Kaminer I. Demonstration of Near-field THz Spectroscopy Using Ultrafast Electron Microscopy. In Conference on Lasers and Electro-Optics; Kang J., , Eds.; Optica Publishing Group, 2021; paper SW2K.4.

[ref50] Gargiulo S.; Madan I.; Barantani F.; Berruto G.; Yannai M.; Dias E. J. C.; Dahan R.; Kaminer I.; Vanacore G. M.; García de Abajo F. J.; Carbone F. Charge Dynamics Electron Microscopy. In Conference on Lasers and Electro-Optics; Kang J., , Eds.; Optica Publishing Group, 2021; paper FM1O.3.

