# Supplementary material for: Charge Dynamics Electron Microscopy: Nanoscale Imaging of Femtosecond Plasma Dynamics
Source: ACS Nano. 2023 Feb 13;17(4):3657–65. doi: 10.1021/acsnano.2c10482 (PMC9979644; doi:10.1021/acsnano.2c10482)
Supplement: Supplementary file 2 — nn2c10482_si_002.pdf [file nn2c10482_si_002.pdf]

# Charge dynamics electron microscopy: nanoscale imaging of femtosecond plasma dynamics

– SUPPORTING INFORMATION–

Ivan Madan,[1,*] Eduardo J. C. Dias,[2,*] Simone Gargiulo,[1,*] Francesco Barantani,[1,3,*] Michael Yannai,[4] Gabriele Berruto,[1] Thomas LaGrange,[1] Luca Piazza,[1] Tom T. A. Lummen,[5] Raphael Dahan,[4] Ido Kaminer,[4] Giovanni Maria Vanacore,[6] F. Javier García de Abajo,[2,7] and Fabrizio Carbone[1,†]

[1]*Institute of Physics, École Polytechnique Fédérale de Lausanne, Lausanne, 1015, Switzerland*
[2]*ICFO-Institut de Ciencies Fotoniques, The Barcelona Institute of Science and Technology, 08860 Castelldefels (Barcelona), Spain*
[3]*Department of Quantum Matter Physics, University of Geneva, 1211 Geneva, Switzerland*
[4]*Department of Electrical and Computer Engineering, Technion, Haifa 32000, Israel*
[5]*BSSE Single Cell Facility, ETH Zurich, Mattenstrasse 26, 4058 Basel, Switzerland*
[6]*Department of Materials Science, University of Milano-Bicocca, Via Cozzi 55, 20126 Milano, Italy*
[7]*ICREA-Institució Catalana de Recerca i Estudis Avançats, Passeig Lluís Companys 23, 08010 Barcelona, Spain*

**Contents**

**S1. Numerical simulations** S1
A. Surface temperature dynamics S1
B. Electronic emission S2
1. Thermionic emission S3
2. Three-photon photoemission S3
C. Charge dynamics S3
D. Energy variation of the probe electron S4

**References** S8

## S1. NUMERICAL SIMULATIONS

In this section, we summarize the details of the theoretical model used to simulate the experiment, leading to the results presented in the main text.

### A. Surface temperature dynamics

The electron temperature dynamics in a copper surface is calculated using the two-temperature model (TTM) [1, 2]. Assuming that the lattice temperature is virtually unchanged (due to its much higher heat capacity), this model can be written in the following simplified form:

$$c_{\rm e}\frac{\partial T}{\partial t} = P^{\rm abs}(z,t) + \nabla\cdot[\kappa_{\rm e}\nabla T] - G_{\rm e}(T-T_0), \tag{S1}$$

where $T(z,t)$ is the electron temperature at position $z$ and time $t$, $T_0$ is the ambient (and lattice) temperature, $c_{\rm e}$ it the heat capacity of the metal (calculated from its electronic density of states [2, 3]), $\kappa_{\rm e}$ is its thermal conductivity [4], $G_{\rm e}$ describes the electron-phonon coupling [5], and $P^{\rm abs}(z,t)$ is the power density absorbed from the external illumination. Assuming a semi-infinite metallic layer at $z<0$ with complex permittivity $\epsilon$ under illumination by a quasi-monochromatic pulse plane wave of central frequency $\omega_0$, peaked at time $t_0$, with FWHM duration $\Delta$, and fluence $F_0$, the absorbed power at the near-the-surface region is given by

$$P^{\rm abs}(z,t) = I_0 k_0 {\rm Im}\{\epsilon\}|\tau|^2 {\rm e}^{2{\rm Im}\{\epsilon\}k_0 z}{\rm e}^{-4\log(2)(t-t_0)^2/\Delta^2}, \tag{S2}$$

*These authors contributed equally.
†To whom correspondence should be addressed: fabrizio.carbone@epfl.ch

where $k_0 = 2\pi c/\omega_0$, $I_0 = 2\sqrt{\log(2)}F_0/(\pi\Delta)$ is the peak intensity and $|\tau|^2$ is the transmittance coefficient across the surface. The latter is determined by using a boundary-element method (BEM)[6, 7] to calculate the field enhancement distribution on the inner surface of an infinitely long copper bar with the same geometrical parameters as in the experiment, tilted 45° relative to the illumination direction (Fig. S1a). From the results above, we construct a map of the surface temperature $T(s,t)$ of the copper bar as a function of time $t$ and surface position $s$ (parameterized as shown in Fig. S1a) for different pump-fluence values (Fig. S1d,e).

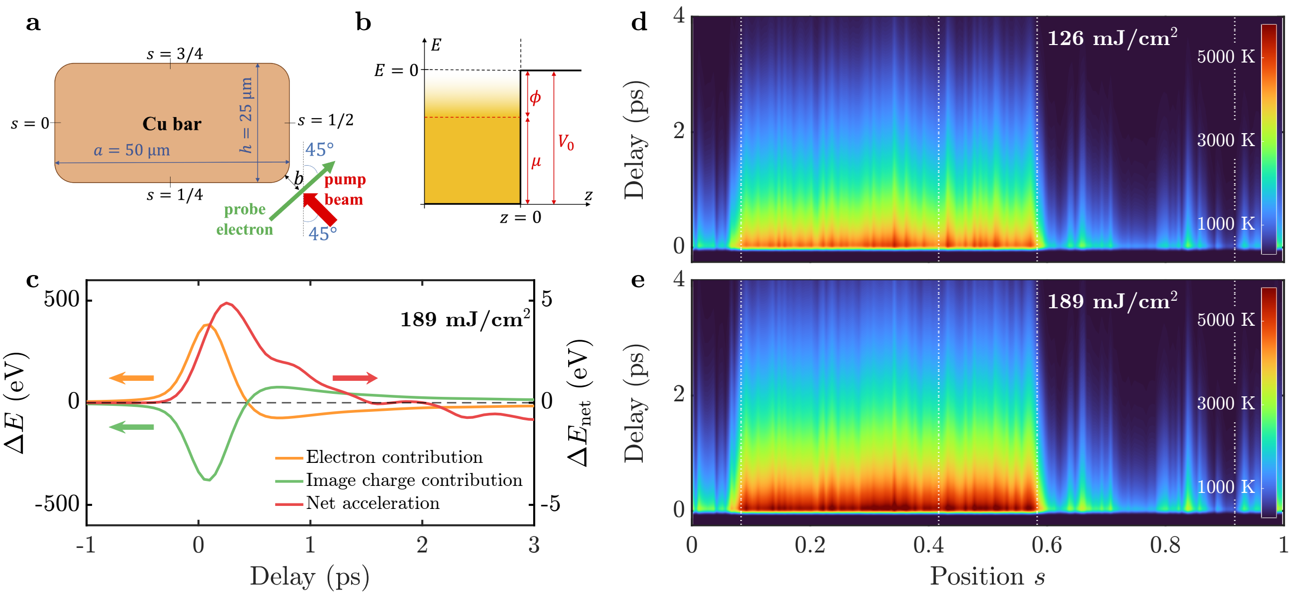


FIG. S1: **Numerical simulation details.** **a**, Scheme of the simulated copper bar cross section, where the characteristic lengths are indicated. The variable $s$ represents the surface parameterization coordinate, ranging from 0 to 1 along its perimeter. The pump beam and electron probe directions are also indicated. Also, $b$ denotes the impact parameter associated to the electron trajectory. **b**, Potential barrier associated with the conduction band of the metal and magnitudes involved in the description of the electron emission process. Here, $E$ is the electron energy, $V_0$ is the total barrier height, $\mu$ is the chemical potential, $\phi = V_0 - \mu$ is the work function, and $z$ is the position-dependent direction perpendicular to the surface. **c**, Individual contribution to the probe-electron energy change by the image (green) and emitted (orange) charges, and corresponding net energy change (red, right vertical scale), all for an impact parameter $b$=1 $\mu$m. **d**, **e**, Spatiotemporal map of the electron temperature at the copper surface upon pump laser excitation, for two fluence values (126 and 189 mJ/cm$^2$). The dashed vertical lines correspond to the parameterized position of the bar corners.

## B. Electronic emission

We assume that the electron emission from the hot copper surface originates mainly in two different processes: (1) direct thermionic emission and (2) three-photon photoemission. The former occurs while the copper surface remains hot (*i.e.*, in a ps timescale, as inferred from Fig. S1d,e), which allows some of the surface electrons with thermally-heightened energy to overcome the potential barrier (see Fig. S1b) and escape. The latter occurs only during the pumping time (< 100 fs) and is due to the absorption of three photons by one electron, providing it with enough energy to overcome the potential barrier. The total electronic emission density per unit of time and surface area as a function of the local and instantaneous electron temperature $T$ is hence given by

$$\mathcal{P}(T) = \mathcal{P}_{\rm thermionic}(T) + \mathcal{P}_{\rm 3ppe}(T), \tag{S3}$$

where $\mathcal{P}_{\rm thermionic}$ and $\mathcal{P}_{\rm 3ppe}$ are the respective contributions from each of the two emission processes mentioned above and described below.

We note that this model neglects other possible emission channels, such as electron tunneling. This mechanism is relevant when the dimensionless Keldysh parameter $\eta$ [8, 9] is $\ll 1$. Under the illumination conditions used in this work, we obtain $\eta \sim 10 - 20$, and hence we neglect it. Additional mechanisms can also take place such as, for example, photoemission of high-energy non-thermal electrons that are not considered in the TTM, but we neglect them because we expect their effect to be a small correction to the emission by the two dominant mechanisms mentioned above.

### 1. *Thermionic emission*

We describe the copper surface as a step potential with a total barrier energy $V_0$, as depicted in Fig. S1b. Conduction electrons obey the Fermi-Dirac distribution

$$f_{\mu,T}(E) = \frac{1}{\mathrm{e}^{-(E+V_0-\mu)/k_\mathrm{B}T}+1}, \tag{S4}$$

where $E$ is the electron energy, $T$ is the electron temperature, and $\mu$ is the temperature-dependent chemical potential. The work function is hence $\phi = V_0 - \mu$.

The probability per unit area $P_\mathrm{thermionic}$ of emitting an electron across the barrier due to thermionic emission is given by

$$P_\mathrm{thermionic} = \frac{2}{A}\sum_{\mathbf{k}_\parallel k'_\mathrm{z}} \mathcal{T}(E_\perp) f_{\mu,T}(E)(1/\tau)\Theta(E), \tag{S5}$$

where $\mathbf{k} = \mathbf{k}_\parallel + k'_\mathrm{z}\hat{\mathbf{z}}$ is the electron wave vector, $E = \hbar^2k^2/2m^*$ is the electron energy (measured with respect to the vacuum level, see Fig. S1b), $m^*$ is the effective mass of the copper electrons, $\Theta(E)$ is the step function, the factor of 2 accounts for spin degeneracy, $A$ is the surface area, $E_\perp = E\cos^2\theta$ is the electron energy perpendicular to the surface, $\theta$ is the emission angle with respect to the normal direction, $\mathcal{T}$ is the transmittance across the energy barrier given by

$$\mathcal{T}(E) = \frac{4\sqrt{E}\sqrt{E+V_0}}{(\sqrt{E}+\sqrt{E+V_0})^2}, \tag{S6}$$

$\tau = 2L/v$ is the time between two consecutive collisions on either side of the metal (spanning a total width $L$ used for quantization), with $v = \hbar k'_\mathrm{z}/m^*$.

Transforming the sums in Eq. (S5) into integrals by performing the customary substitutions $\sum_{\mathbf{k}_\parallel} \to A/(2\pi)^2\int d\mathbf{k}_\parallel$ and $\sum_{k'_\mathrm{z}} \to L/2\pi\int dk'_\mathrm{z}$, we can simplify the previous equation to $P_\mathrm{thermionic} = \int dE \int d\theta\, \mathcal{P}_\mathrm{thermionic}(T)\Theta(E)$, where

$$\mathcal{P}_\mathrm{thermionic}(T) = \frac{m^*}{2\pi^2\hbar^3} E\cos\theta f_{E,T}\mathcal{T}(E\cos^2\theta) \tag{S7}$$

represents the probability of thermionically emitting an electron of energy $E$ along an angle $\theta$ at a surface position with temperature $T$.

### 2. *Three-photon photoemission*

The three-photon photoemission is calculated using the well-known Fowler-Dubridge model, according to which the emission probability is given by [10]

$$\mathcal{P}_\mathrm{3ppe}(T) = a_3 A \left(\frac{e}{\hbar\omega}\right)^3 I_\mathrm{abs}^3 T^2 F\left(\frac{3\hbar\omega - e\phi}{k_\mathrm{B}T}\right), \tag{S8}$$

where $A = 120$ A/cm$^2$K$^2$ is the Richardson constant, $e > 0$ is the electron charge, $\omega$ is the pump frequency, $I_\mathrm{abs}$ is the absorbed power density, $T$ is the local surface temperature, $F(x)$ is the Fowler function [10], and $a_3$ represents the likelihood of the emission, which is a measured parameter that depends on the material and pump characteristics. We set $a_3 \sim 0.5\times10^{-35}$ cm$^6$/A$^3$ as a suitable value for this parameter to explain our experiments. This is one order of magnitude lower than previously reported estimates for three-photon photoemission in copper [11, 12], which could be explained by saturation in the emission due to the high pump fluences used in this work. A saturation effect on the photoemission current has been reported before in tungsten samples under strong fluence illumination ($\gtrsim 10$ mJ/cm$^2$), which has been attributed to charge saturation from space-charge accumulation outside the metal [13] Also, the presence of an oxide layer on the outer copper surface could contribute to this reduction of $a_3$. Additionally, this parameter may be regarded as a way to include an effective correction to the calculated values of the temperature and potential barrier height.

## C. Charge dynamics

Using the functions in Eq. (S7) and (S8) together with the data from Fig. S1e,f, one can simulate the density of electrons $\rho_\mathrm{e}(\mathbf{R},t)$ photoemitted from the surface of the copper bar as a function of time, normalized per unit

length along the extension of the bar. Importantly, the emission of electrons leaves an unbalance of charges inside the copper, effectively resulting in the generation of a positive surface image charge density $\sigma_{\rm s}$ along the side length of the copper bar, which depends on the distribution of electrons on the outside. This redistribution of conduction electrons inside the metal rapidly follows the emitted electron dynamics, driven by internal screening that takes a time of the order of the plasmon optical period (a few fs) to be established. In our simulations, we calculate the surface density of these image screening charges by treating the metal as a perfect conductor at constant potential in the presence of the emitted electrons, imposing total charge neutrality, and using a boundary-element-method approach.

Numerically, the henceforth described electronic emission is implemented by discretizing both the time and the copper surface position (here multiplexed by the label $j$), and, at each time step, calculating the corresponding electronic emission probability $P_j$ (using Eqs. (S7) and (S8)) as well as the average velocity $\mathbf{v}_j$ associated with electrons produced by each of the two processes described above. For simplicity, we assume that the emitted electrons are ejected primarily along the perpendicular direction with respect to the metallic surface. While we loosely refer to the emitted particles as electrons, in this model they rather correspond to effective electron ensembles with charge $q_j = -eP_j$ and mass $m_j = m_{\rm e}P_j$.

Next, after each new time step $\delta t$, we calculate the Coulomb force $\mathbf{f}_j$ exerted at each emitted electron $j$ by the remaining electrons and surface charges, and evolve its position and velocity accordingly, that is,

$$\mathbf{r}_j(t+\delta t) = \mathbf{r}_j(t) + \mathbf{v}_j(t)\delta t, \tag{S9}$$

$$\mathbf{v}_j(t+\delta t) = \mathbf{v}_j(t) + \mathbf{a}_j(t)\delta t, \tag{S10}$$

where $\mathbf{a}_j = \mathbf{f}_j/m_j$. The reabsorption probability by the surface is modeled by introducing a probability $\mathcal{R} = 1 - \mathcal{T}(E_\perp) < 1$ (see Eq. (S6)) that reduces the fraction of electrons reflected upon collision with the surface; electrons that are not absorbed are taken to be specularly reflected and their effective weight is renormalized as $P_j(t+\delta t) = \mathcal{R}P_j(t)$.

In Fig. S2a, we show the behavior of the total number of emitted charges and free space charges as a function of time, the latter is characterized by a strong increase right after the pump pulse, and a slow decrease over a few ps due to the progressive electron reabsorption by the surface. This behavior is clear when analyzing Fig. S2c, which shows several snapshots of the spatial distribution of emitted and surface charge densities as a function of time. Figure S2B shows the distribution of surface charge density along the copper surface for a selected time instant, and it is characterized by a strong accumulation over the surfaces facing directly the pump incidence (see Fig. S1a).

### D. Energy variation of the probe electron

We now consider the process of probing the system by means of an e-beam electron moving at velocity $\mathbf{v}_{\rm e}$, as indicated by the green arrow in Fig. S1a. Taking the nearest point to the copper bar along the electron trajectory as $\mathbf{r}_0$, we describe the trajectory of the electron as $\mathbf{r}_{\rm e}(t) = \mathbf{r}_0 + \mathbf{v}_{\rm e}(t+\tau)$, where $\tau$ is the delay of the probe electron with respect to the laser pump.

The energy variation of the probe electron along its trajectory is given by

$$\Delta E = -e \int d\mathbf{r}_{\rm e} \cdot \mathcal{E}(\mathbf{r}_{\rm e}, t) = -e\mathbf{v}_{\rm e} \cdot \int_{-\infty}^{\infty} dt\, \mathcal{E}[\mathbf{r}_{\rm e}(t), t] \equiv \int_{-\infty}^{\infty} dt\, \Gamma(t), \tag{S11}$$

where we define $\Gamma(t)$ as the rate of electron energy variation. Ignoring the effect of the currents produced by the photoemitted electrons (due to their very low drift velocity), this electric field is produced by both the photoemitted electrons, $\mathcal{E}_{\rm e}$, and the screening surface charges, $\mathcal{E}_{\rm h}$, which give rise to two different contributions for the electron energy variation rate, $\Gamma_{\rm e}(t)$ and $\Gamma_{\rm h}(t)$, respectively. More precisely,

$$\Gamma_{\rm e}(t) = 2e^2 \int d^2\mathbf{R}\, \rho_{\rm e}(\mathbf{R}, t_{\rm r}) \frac{\mathbf{v}_{\rm e} \cdot (\mathbf{r}_{\rm e}(t) - \mathbf{R})}{|\mathbf{r}_{\rm e}(t) - \mathbf{R}|^2}, \tag{S12}$$

$$\Gamma_{\rm h}(t) = -2Le^2 \int_0^1 ds\, \sigma_{\rm s}(s, t_{\rm r}) \frac{\mathbf{v}_{\rm e} \cdot (\mathbf{r}_{\rm e}(t) - \mathbf{r}_s)}{|\mathbf{r}_{\rm e}(t) - \mathbf{r}_s|^2}, \tag{S13}$$

where $\mathbf{R}$ denotes the coordinates of the emitted electrons, $\mathbf{r}_s$ stands for the coordinates of the point along the surface corresponding to the parameter $s$, $\rho_{\rm e}$ and $\sigma_{\rm s}$ are the density of photoemitted electrons and screening surface charges, respectively (see Fig. S2c), $L$ is the perimeter of the copper bar cross section, and $t_{\rm r}$ is the retarded time defined as $t - |\mathbf{r}_{\rm e}(t) - \mathbf{R}|/c$ and $t - |\mathbf{r}_{\rm e}(t) - \mathbf{r}_s|/c$ respectively. In order to account for the electron wave packet time duration $\Delta t_{\rm e} \sim 600$ fs, we correct Eqs. (S12) and (S13) by performing a convolution with a normalized Gaussian function $G(t'-t)$ centered at $t$ with FWHM $\Delta t_{\rm e}$ and adjusting the position of the electron

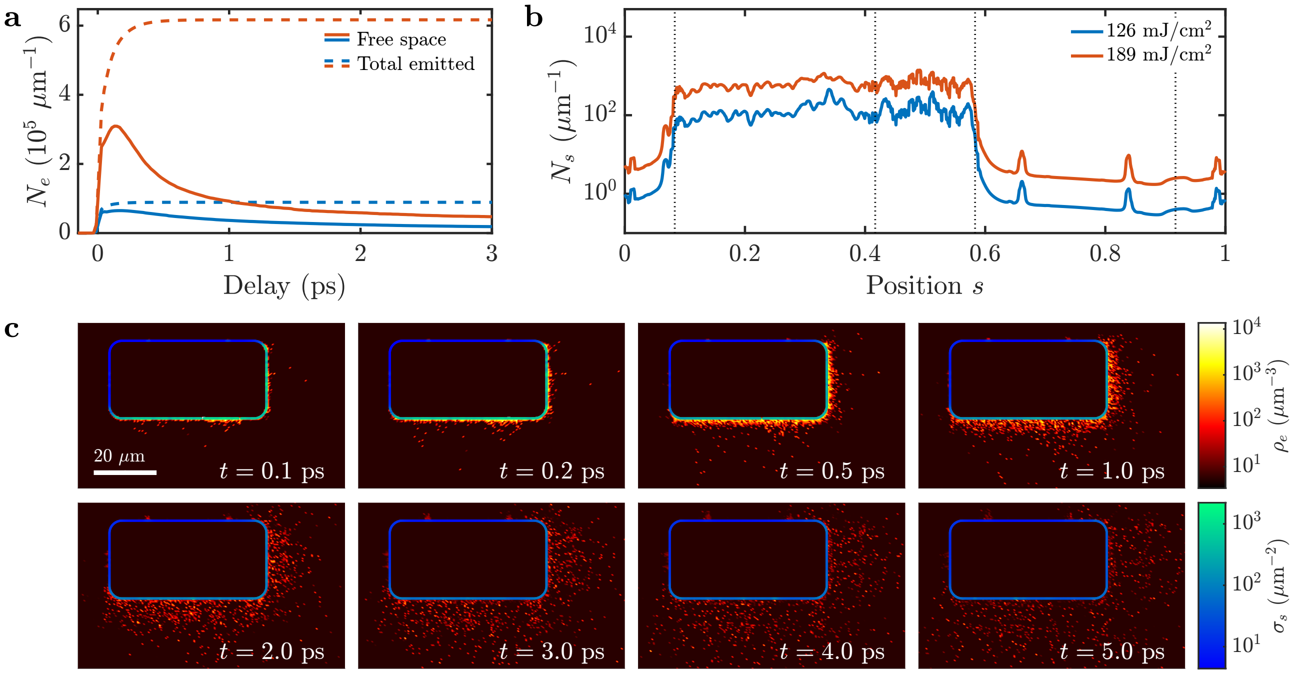


FIG. S2: **Plasma charge dynamics.** **a**, Dynamics of the integrated density of emitted charges in free space (solid curves) and total charges (*i.e.*, accumulated emission, dashed curves) for two fluence values (126 and 189 mJ/cm$^2$) as a function of delay defined with respect to the center of the pump laser pulse. **b**, Spatial distribution along the surface of the image charge density (at the time instant when the integrated density is maximum), for the same fluences as in (a). The dashed vertical lines correspond to the parameterized position of the bar corners. **c**, Snapshots at selected delay instants (see labels) of the thermally emitted electron distribution $\rho_e$, and associated surface density distribution $\sigma_s$ along the bar surface.

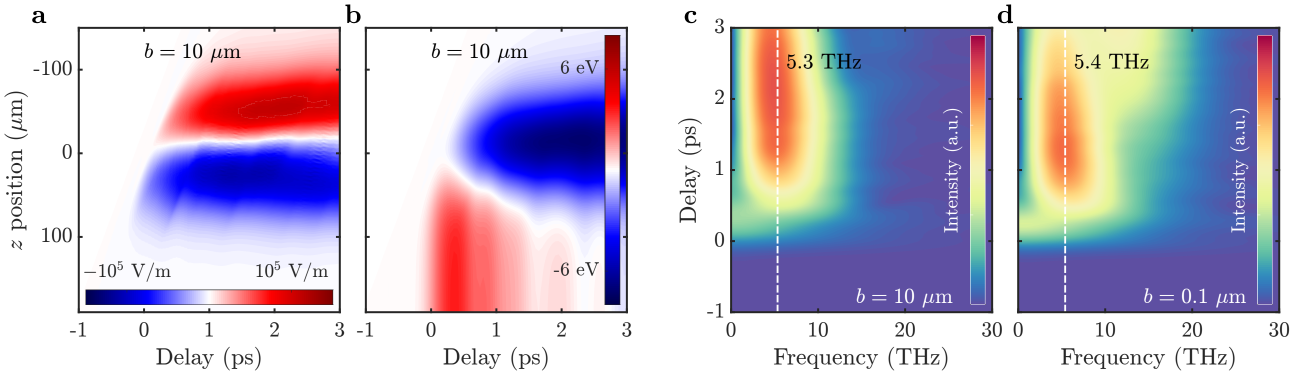


FIG. S3: **Energy variation of the probe electron.** **a**, Electric field at the frame of the probe electron along its trajectory as a function of position along the trajectory (vertical axis, with 0 representing the point closest to the copper bar) and delay between the e-beam and pump-laser pulses. The electric field is chosen to be positive if it points in the same direction as the electron velocity. **b**, Analogous to panel a, but showing the electron energy variation along its motion. **c**, **d** Fourier transform of the temporal profile of the electric field for impact parameters of 10 $\mu$m and 0.1 $\mu$m (see labels). All the results are calculated for an incident fluence of 189 mJ/cm$^2$. **a** and **b** are calculated for an impact parameter of 10 $\mu$m.

accordingly, ie., replacing $\Gamma(t)$ in Eq. (S11) by

$$\Gamma^{\rm av}(t) = \int_{-\infty}^{\infty} dt' \; G(t'-t)\Gamma(t)|_{\mathbf{r}_{\rm e}(t)\to\mathbf{r}_{\rm e}(t')}\,, \tag{S14}$$

where $G(t) = (2/\Delta t_{\rm e})\sqrt{\log(2)/\pi}\,{\rm e}^{-4\log(2)t^2/\Delta t_{\rm e}^2}$. The broadening of the spectrum of the energy variation seen in Fig. 2g of the main text is calculated by sweeping all values of $\Delta t = t' - t$ and assigning to each value of the energy variation given by $\Delta E = \int_{-\infty}^{\infty} dt \; \Gamma(t)|_{\mathbf{r}_{\rm e}(t)\to\mathbf{r}_{\rm e}(t+\Delta t)}$ a corresponding weight $G(\Delta t)$ (analogously to the number of counts in the experiment).

A graphical representation of the net electric field acting on the probe electron along its trajectory is presented

in Fig. 2b in the main text and Fig. S3a below, for two different impact parameters (0.1 and 10 $\mu$m, respectively). The resulting accumulated energy variation is shown in Fig. 2f and Fig. S3b, respectively. These panels show that, for both impact parameters, if the delay between the electron and the pulse is $\gtrsim 0.5$ ps, the probe electron experiences in a first instance a significant deceleration as it approaches the emitted electron cloud (red region in Fig. 2b and Fig. S3a), and a subsequent acceleration after it crosses the cloud (blue region in the same panels). If the delay is large enough, the deceleration and acceleration compensate each other, resulting in a negligible energy variation (see Fig. 2f and Fig. S3f for large position values). In contrast, if the delay is small enough ($\lesssim 0.5$ ps), the deceleration of the probe electron is suppressed because it passes by the cloud region before there is a significant number of emitted cloud electrons, resulting in a large net energy gain, as observed in the experiments (corresponding to the bright red region in Fig. 2f and Fig. S3b). The electric field and energy variation dynamics described above can be observed in Movie S1, where probe electrons for different time delays and impact parameters are overlaid with the spatiotemporal plasma evolution. In the right panel, their instantaneous electric field and energy variation are plotted as a function of the position along the trajectory, for each time instant.

In Fig. S3c,d, we show the spectral profile of the electric field experienced by the probe electron for two values of the impact parameter (0.1 and 10 $\mu$m, respectively), obtained by taking the Fourier transform of the temporal profile of the field presented in Fig. 2b and Fig. S3a as a function of time delay, that is,

$$\tilde{\mathcal{E}}(\omega) = \int_{-\infty}^{\infty} dt\, \mathcal{E}[\mathbf{r}_\mathrm{e}(t), t]\mathrm{e}^{-\mathrm{i}\omega t}. \tag{S15}$$

For delays $\gtrsim 0.5$ ps, the electric field exhibits a clear peak centered around 5 THz for both impact parameters. In contrast, for delays $\lesssim 0.5$ ps, the peak frequency is lower because, in such case, the electron only undergoes acceleration, and therefore, the field variation is milder.

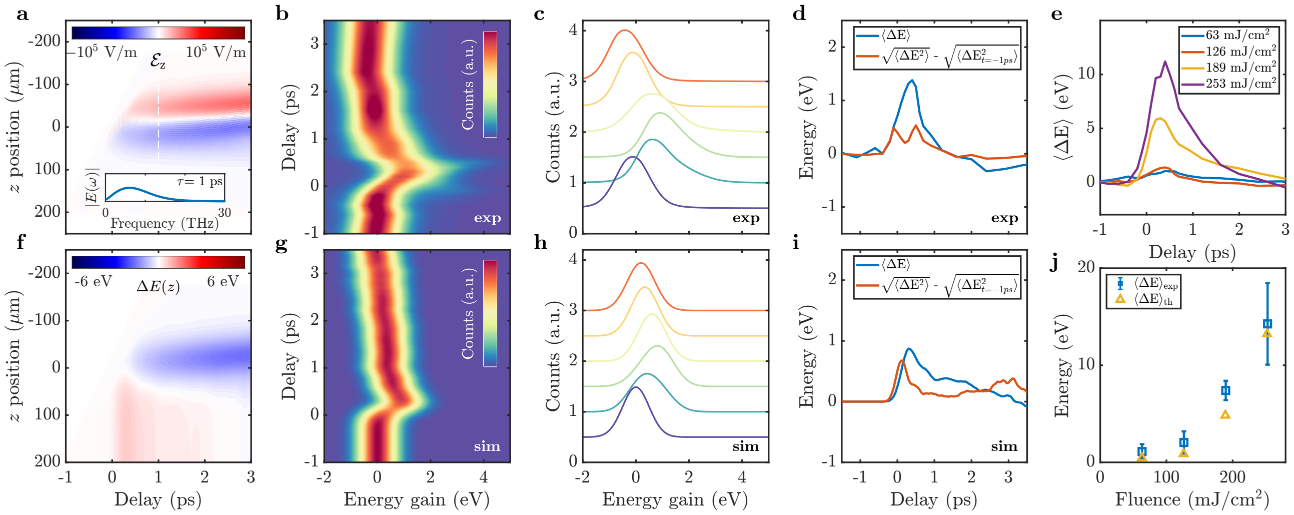


FIG. S4: **Ultrafast e-beam interactions in CDEM under 126 mJ/cm$^2$ laser pulse.** **a**, Simulated electric field experienced by the e-beam as a function of delay and position along the trajectory ($z = 0$ corresponding to the e-beam leveled with the tip of the metal corner in Fig. 1) for an e-beam–surface separation of 100 nm. The inset shows the Fourier-transformed electric-field amplitude at a delay of 1 ps (white dashed line), peaking at 6 THz. **b**, Transmitted electron spectra as a function of laser-e-beam delay for 100-nm e-beam-surface separation. **c**, Profiles extracted from **c** at selected delays (see labels). **d**, Variation of the average e-beam energy and spectrum variance as a function of delay. **e**, Variation of the average e-beam energy for different fluences. **f**, Calculated modification of e-beam energy $\Delta E(z)$ as a function of both electron position $z$ and delay between optical and electron pulses as it experiences the effect of the electric field $\mathcal{E}_\mathrm{z}$ plotted in **b**. **g**, **h**, **i** Numerical simulations based on microscopic theory corresponding to the conditions in **b**, **c**, and **d** respectively. **j** Fluence dependence of the maximum average e-beam energy from experiments (blue square) and simulations (yellow triangles).

MOVIE S1: **Time evolution of the electron energy variation Left**: spatiotemporal evolution of the plasma density (in logarithmic scale), and real-time trajectory of probe electrons with varying impact parameter (1.0 and 10.0 $\mu$m, respectively represented by cyan and pink) and time delay (0.5 and 2.0 ps). The perimeter of the copper bar is marked by the dashed yellow curve. The extension of the probe electrons along their trajectory is due to the pulse duration (600 fs FWHM). **Right**: instantaneous electric field $\mathcal{E}$ along the trajectory (top) and corresponding energy variation $\Delta E$ until that instant (bottom), for the electron trajectories presented on the left panel (0.5 and 2.0 ps delay times respectively represented by solid and dashed curves). In each moment, the time instant is indicated, with $t = 0$ corresponding to the instant when the pump pulse peak impinges on the copper bar. All data is for an incident fluence of 189 mJ/cm$^2$.

## References


[1] Anisimov, S.; Kapeliovich, B.; Perelman, T., et al. Electron Emission from Metal Surfaces Exposed to Ultrashort Laser Pulses. *Zh. Eksp. Teor. Fiz* **1974**, *66*, 375–377.

[2] Block, A.; Liebel, M.; Yu, R.; Spector, R.; Sivan, Y.; García de Abajo, F. J.; van Hulst, N. F. Tracking Ultrafast Hot-Electron Diffusion in Space and Time by Ultrafast Thermomodulation Microscopy. *Sci. Adv.* **2019**, *5*, eaav8965.

[3] Electron Density of States for FCC Copper. `https://lampx.tugraz.at/~hadley/ss1/materials/dos/fccCu_dos.html`, Accessed: 2022-03-09.

[4] Thermal Conductivity of Copper. `https://www.efunda.com/materials/elements/TC_Table.cfm?Element_ID=Cu`, Accessed: 2022-03-09.

[5] Lin, Z.; Zhigilei, L. V.; Celli, V. Electron-Phonon Coupling and Electron Heat Capacity of Metals under Conditions of Strong Electron-Phonon Nonequilibrium. *Physical Review B* **2008**, *77*, 075133.

[6] García de Abajo, F. J.; Howie, A. Relativistic Electron Energy Loss and Electron-Induced Photon Emission in Inhomogeneous Dielectrics. *Physical Review Letters* **1998**, *80*, 5180–5183.

[7] García de Abajo, F. J.; Howie, A. Retarded Field Calculation of Electron Energy Loss in Inhomogeneous Dielectrics. *Physical Review B* **2002**, *65*, 115418.

[8] Dombi, P.; Pápa, Z.; Vogelsang, J.; Yalunin, S. V.; Sivis, M.; Herink, G.; Schäfer, S.; Groß, P.; Ropers, C.; Lienau, C. Strong-Field Nano-Optics. *Reviews of Modern Physics* **2020**, *92*, 025003.

[9] Keldysh, L. V. Ionization in the Field of a Strong Electromagnetic Wave. *Sov. Phys. JETP* **1965**, *20*, 1307–1314.

[10] Ferrini, G.; Banfi, F.; Giannetti, C.; Parmigiani, F. Non-Linear Electron Photoemission from Metals with Ultrashort Pulses. *Nuclear Instruments and Methods in Physics Research Section A: Accelerators, Spectrometers, Detectors and Associated Equipment* **2009**, *601*, 123–131.

[11] Musumeci, P.; Cultrera, L.; Ferrario, M.; Filippetto, D.; Gatti, G.; Gutierrez, M.; Moody, J.; Moore, N.; Rosenzweig, J.; Scoby, C., et al. Multiphoton Photoemission from a Copper Cathode Illuminated by Ultrashort Laser Pulses in an RF Photoinjector. *Physical Review Letters* **2010**, *104*, 084801.

[12] Tsang, T.; Srinivasan-Rao, T.; Fischer, J. Surface-Plasmon Field-Enhanced Multiphoton Photoelectric Emission from Metal Films. *Physical Review B* **1991**, *43*, 8870.

[13] Fujimoto, J.; Liu, J.; Ippen, E.; Bloembergen, N. Femtosecond Laser Interaction with Metallic Tungsten and Nonequilibrium Electron and Lattice Temperatures. *Physical Review Letters* **1984**, *53*, 1837.
